# The effect of corticosteroid injection in the treatment of greater trochanter pain syndrome: a systematic review and meta-analysis of randomized controlled trials

**DOI:** 10.1186/s13018-022-03175-5

**Published:** 2022-05-21

**Authors:** Yule Wang, Kaijin Wang, Yiling Qin, Sanrong Wang, Botao Tan, Lang Jia, Gongwei Jia, Lingchuan Niu

**Affiliations:** 1 Department of Rehabilitation, The Second Affiliated Hospital of Chongqing Medical University, Chongqing, 400010 China; 2 Department of Respiratory Medicine, Jiangjin District Central Hospital, Chongqing, 400010 China; 3 Department of Pediatrics, Angel Maternity Hospital, Chongqing, 400010 China

**Keywords:** Greater trochanter pain syndrome, Corticosteroid injection, Pain, Function, Meta-analysis

## Abstract

**Background:**

corticosteroid injection (CSI) has been used to treat greater trochanter pain syndrome (GTPS) for many years. However, so far, the efficacy of CSI in the treatment of GTPS is still controversial. Therefore, the aim of this review is to evaluate the effectiveness of CSI in comparison with sham intervention, nature history, usual care, platelet-rich plasma (PRP), physiotherapy/exercise therapy, dry needling, or other nonsurgical treatment for improvements in pain and function in GTPS.

**Methods:**

PubMed (Medline), Embase, Cochrane Library were searched from their inception until April 2021. Randomized controlled trails (RCTs) comparing CSI to nonsurgical treatment were included. Data on the effect of CSI on pain and function were extracted and checked by two review authors independently. The treatment effect was analyzed in the short term, medium term, and long term.

**Results:**

Eight RCTs (764 patients) were included. This review suggests CSI may be superior to usual care and ‘wait and see,’ ESWT, but may not be superior to exercise, PRP, dry needling, and sham intervention in short-term pain or function improvement. In terms of medium-term pain or function improvement, CSI may be superior to usual care and ‘wait and see,’ but may not be superior to PRP. In terms of long-term pain or function improvement, CSI may be inferior to PRP and ESWT, but it may be superior to usual care and ‘wait and see’ at 12 months.

**Conclusions:**

Due to the small sample size and lack of sufficient clinical studies, current evidence is equivocal regarding the efficacy of CSI in the treatment of GTPS. Considering the limitations, more large-sample and high-quality RCTs are needed to prove the therapeutic effect of CSI on GTPS.

***Trial registration*:**

PROSPERO registration number: CRD42021247991. Registered 09 May 2021.

**Supplementary Information:**

The online version contains supplementary material available at 10.1186/s13018-022-03175-5.

## Background

Greater trochanteric pain syndrome (GTPS) is a chronic lateral pain of the hip joint, which has a significant negative impact on function, sleep, and quality of life [1]. It refers to the general term for a series of diseases that cause local pain symptoms due to lesions or injuries of the tissue structure attached to the greater trochanter of the femur. The main causes include inflammation or tear of the gluteus medius and gluteus minimus tendons, and bursitis around the greater trochanter [2, 3]. The incidence of GTPS in the population is 10%-25%, and it mainly affects middle-aged women(40–60y) [4, 5].

Conservative treatment is the first-line approach for GTPS, including ﻿correction of gait disorders, relative rest, cold and heat, stretching and strengthening, physiotherapy, drugs (e.g., NSAIDs, opioids, antidepressants, topical treatments), corticosteroid injection (CSI), extracorporeal shockwave therapy (ESWT) [6–9]. Some intractable ones require surgical treatment [2, 10].

CSI has been used to treat GTPS for many years [11]. Some studies found that CSI can effectively relieve the pain of GTPS [12–14]. A 2011 systematic review evaluated efficacy of treatment of GTPS, including CSI, and found that CSI could relieve pain and return to activity in 49–100% of patients [15]. A systematic review in 2012 assessed conservative and surgical treatments of GTPS, including CSI, and found that the best treatment for GTPS could not be definitively concluded due to poor studies [10]. A 2016 systematic review evaluating conservative treatments for GTPS found that CSI is superior to home training, ESWT, and usual care for up to 3 months [16]. Up to now, the efficacy of CSI in the treatment of GTPS is still controversial, and the previous systematic review did not use meta-analysis. Therefore, we conducted this meta-analysis and systematic review to evaluate the effect of CSI in comparison with sham intervention, nature history, usual care, platelet-rich plasma (PRP), physiotherapy/exercise therapy, dry needling, or other nonsurgical treatment for improvements in pain and function in GTPS.

## Materials and methods

This meta-analysis was registered online in PROSPERO (registration number: CRD42021247991) and was conducted in accordance with the Preferred Reporting Items for Systematic Reviews and Meta-Analyses guidelines [17]. The PRISMA checklist was provided as Additional file 1: PubMed (Medline), Embase, and Cochrane Library were searched up to April 30, 2021. The following search terms were used to retrieve literature: (‘greater trochanter pain syndrome’ or ‘greater trochanteric pain syndrome’ or GTPS or ‘trochanteric bursitis’ or ‘gluteal tendinopathy’) and (corticosteroid or glucocorticoid) and (Injection). In addition, the reference lists of the included articles and relevant systematic reviews were also reviewed for additional studies. The language was limited to English.

All relevant articles were independently screened for inclusion by two co-authors (Y. Q. and K. W.), and the disagreements between them were resolved through discussion. The studies were considered qualified if they met the following inclusion criteria: (1) randomized controlled trails (RCTs), (2) the studies enrolled patients with GTPS, (3) one group used CSI to treat GTPS and the other used sham intervention, nature history, usual care, platelet-rich plasma (PRP), physiotherapy, exercise therapy, dry needling, or other nonsurgical treatment. Nonhuman studies, non-English studies, case series, case reports, cohort studies, review article, comments, conference abstract, unpublished studies were excluded from this review. Studies that included patients under the age of 18, patients with infection, acute trauma, rheumatoid arthritis, and patients who had undergone hip surgery were also excluded.

The data of the included RCTs were independently extracted by the two co-authors (S. W. and B. T.), and the disagreements were resolved through discussion. Details were extracted from each included trial: name of main author, year of publication, country of study, study design, sample size, mean age, average onset, number of patients in each study group, intervention protocol of each study group, outcome type (scales for pain and function), and follow-up time.

The quality of included RCTs was assessed using the Cochrane Collaboration’s Risk of Bias approach [18]. Quality assessment was independently performed by two co-authors (L. J. and G. J.), and the disagreements were resolved through discussion to reach a consensus.

The outcomes of pain and function were categorized as: (1) short term (1 to  ≤ 6 weeks), (2) medium term (6 to ≤ 12 weeks) , or (3) long term (> 12 weeks). The short-term and medium-term outcomes were analyzed using the latest data in each time category. However, since there is no maximum limit in the long-term category, the long-term outcomes were analyzed based on the data of similar follow-up time of included articles.

RevMan 5.4.1 software was used to perform all analysis. The mean difference (MD) or ﻿standardized mean difference (SMD) with 95% confidence intervals (CI) was used for summary statistics. SMD was used when included studies used different scales to measure the same result (pain or function). Chi-square test (Q test) and *I*^2^ statistic were used to evaluate the statistical heterogeneity of the pooled data, and *P* < 0.1 or *I*^2^ value > 50% indicated significant heterogeneity [19]. A random-effects model was employed when there was significant heterogeneity, otherwise, a fixed-effect model was used. The potential influence of small sample sizes study biases was addressed by the risk of bias criterion 'study size' [20]. A study with a sample size of less than 50 participants, between 50 and 200 participants, more than 200 participants was considered at high risk, moderate risk, low risk of small sample bias. A funnel plot was used to assess the likelihood of potential publication bias when more than 10 studies were included [21]. A sensitivity analysis was performed to evaluate the reliability of the findings by removing each study.

## Results

**Fig. 1:**
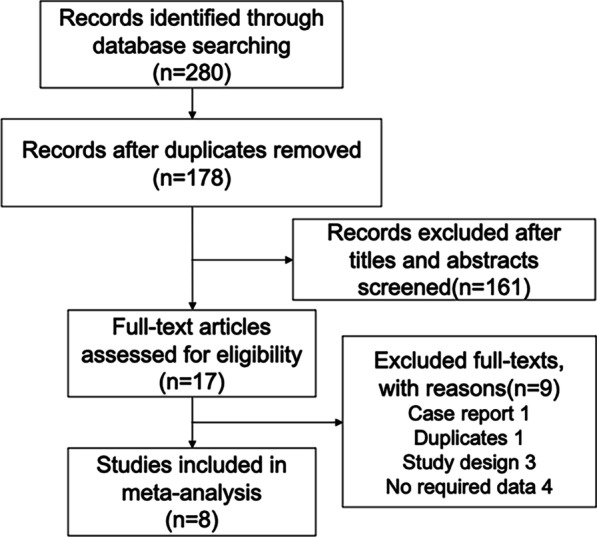
study flow chart

A total of 280 records were retrieved from the literature research, leaving 178 studies after duplicates removed. Then 161 studies were excluded after titles and abstracts screened, and 17 studies were left for full-text screening. Due to case report [22], duplicate [23], no required data [24–27], and study design [28–30], 9 studies were excluded. Finally, 8 RCTs [31–38]were included in our meta-analysis, which included a total of 764 participants. The study flowchart is shown in Fig. 1.

Ten groups of 8 studies assessed the efficacy of CSI relative to other treatments. Of these, three studies, respectively, compared CSI to sham intervention [34], usual care [36], and wait and see [31], two studies compared CSI to exercise intervention [31, 32], one study compared CSI to dry needling [37], three studies compared CSI to PRP [33, 35, 38], and one study compared CSI to SWT [32]. One study had 6 months of following data, but it was partially unblinded at 4 weeks, so we only selected the data for the fourth week for analysis [34]. One study recorded data on both ‘pain at rest’ and ‘pain at activity,’ and we selected ‘pain at activity’ for analysis [36]. At the same time, this study only reported the data at 3-month and 12-month follow-up, while the data for 6 weeks, 6 months, and 9 months were directly shown on the graph. We intercepted the mean and standard error data from the graph and converted the standard error into standard deviation according to the formulae provided by the chapter 7.7.3.2 of Cochrane Handbook for Systematic Reviews of Interventions (ChineseDec2014). The characteristics of the included studies are presented in Table 1.

**Table 1:** Characteristics of the included studies

Study, year, country	Study design	Sample size (n, M/F)	Mean age (years (SD), CSI/control)	Average onset (CSI/control)	Study group (n, CSI/control)	CSI group protocol	Control group protocol	Outcomes	Follow-up
Rompe et al. (2009) Germany	RCT	229 (67/162)	50 (not reported)/ (46 (not reported)/ 47 (not reported))	11 m/ (14 m/15 m)	75/ (76/78)	0.5% Mepivacainmixed with 1 mL of Prednisolone 25 mg	home training/ ESWT	6-point Likert scale, NRS	15 m
Nissen et al. (2018) Switzerland	RCT	46 (7/39)	56.6 (14.6)/ 59.6 (13.1)	≥ 1 m	21/25	4 ml of 1% lidocaine and 1 ml of betamethasone	placebo: 5 ml of sterile saline solution	NRS, 5-point Likert scale, WOMAC, SF-12, Oswestry low back pain questionnaire	6 m
Brinks et al. (2011), Netherlands	RCT	120 (28/92)	57.7 (13.9)/ 54.8 (14.7)	≥ 1w	60/60	40 mg of triamcinolone acetate combined with 1% or 2% lidocaine	usual care: analgesics	7-point Likert scale, NRS, EQ-5D, WOMAC	12 m
Brennan et al. ( 2017), USA	RCT	43 (6/37)	70.1 (11.4)/ 61.3 (16.5)	not reported	22 (25 hips)/ 21 (25 hips)	2 ml methylprednisolone acetate 40 mg/ml; 4 ml 1% lidocaine; 4 ml 0.25% marcaine (10 ml total)	dry needling	NRS, PSFS	6w
Ribeiro et al., (2016), Brazil	RCT	18 (8/10)	49.6 (11.66)/ 50 (17.8)	≥ 3 m	9 (10 hips)/ 9 (10 hips)	4 ml solution of 80 mg of triamcinolone hexacetonide	PRP	FEPS, HHS, WOMAC	60d
Begkas et al. (2020), GRC	RCT	24 (6/18)	not reported	≥ 12w	12/12	4 ml of methylprednisolone (40 mg/ml)	PRP	VAS, HHS	24w
Fitzpatrick et al. (2018), Australia	RCT	80 (8/72)	59.7(not reported)/ 60.3(not reported)	≥ 4 m	40/40	Celestone Chronodose with saline	PRP	mHHS, PASS	12w
Mellor et al. (2018), Australia	RCT	204 (37/167)	55.3 (9.4)/ (54.8 (8.1)/54.5 (9.1))	18 m/ (24 m/24 m)	66/ (69/69)	1 ml betamethasone (5.7 mg/ml) or 1 ml triamcinolone acetonide (40 mg/ml) combined with 2 ml bupivacaine or 1 ml Marcaine	education plus exercise/ wait and see	NRS, VISA-G, PSFS, EQ-5D, PSEQ, PCS, PHQ-9, LHPQ, the Active Australia survey	52w

CSI, Corticosteroid injection; d, Day; ESWT, Extracorporeal shockwave therapy; EQ-5D, European quality of life-5D questionnaire; F, Female; FEPS, Facial expressions pain scale; HHS, The Harris Hip Score; LHPQ, Lateral hip pain questionnaire; M, Male; m, Month; mHHS, The modified Harris Hip Score; NRS, Numerical rating scale; PSFS, Patient-specific functional scale; VAS, Visual analogue scale; PASS, Patient acceptable symptom state; PSEQ, Pain self-efficacy questionnaire; PCS, Pain catastrophising scale; PHQ-9, Patient health questionnaire 9; RCT, Randomized controlled trail; SD, Standard deviation; VISA-G, Victorian Institute of Sport Assessment—gluteal tendinopathy; w, Week; WOMAC, The Western Ontario and McMaster University Osteoarthritis Index

**Fig. 2:**
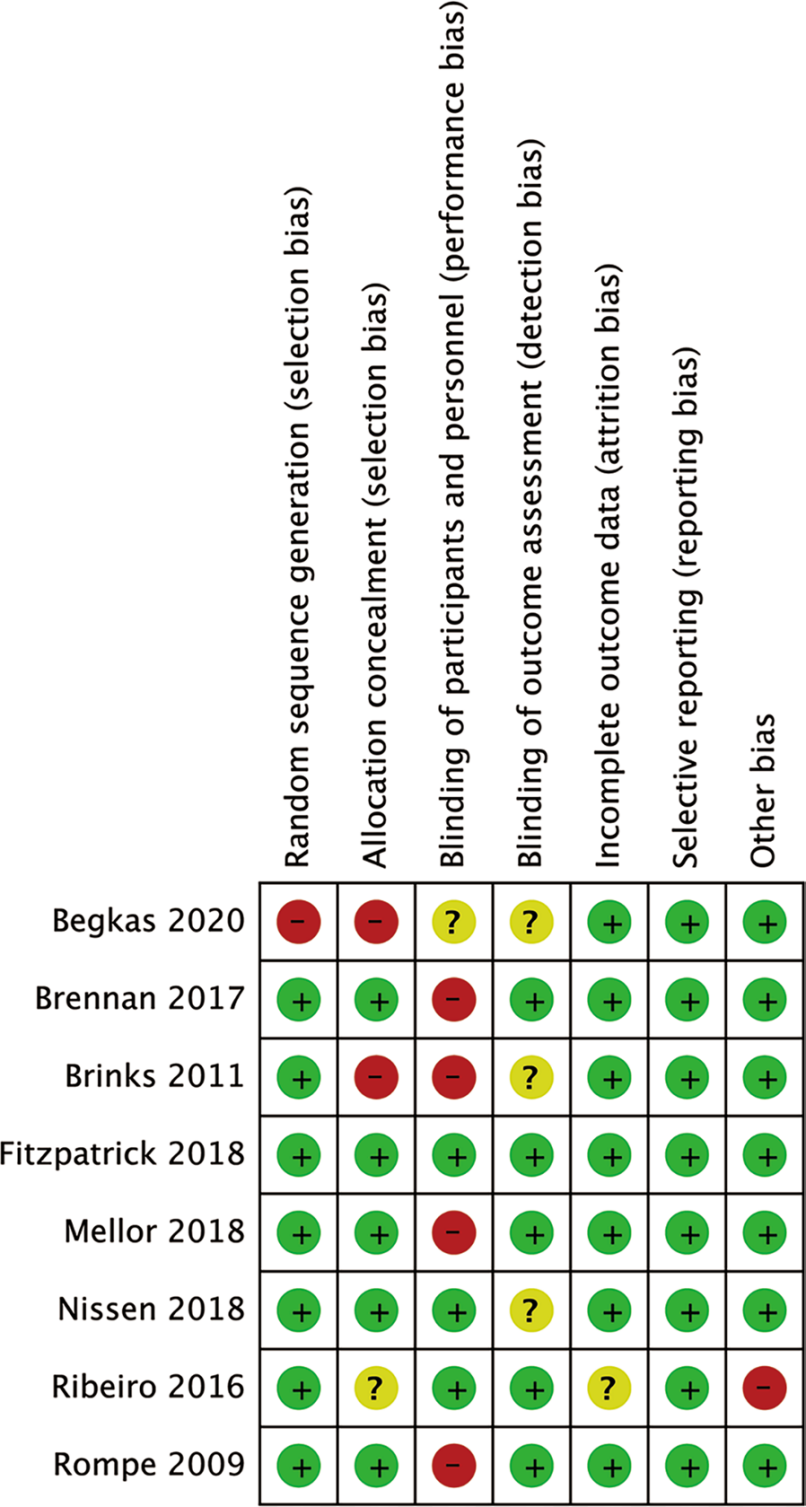
Risk of bias summary in included studies

The risk of bias in included studies is summarized in Fig. 2. The random sequence generation was incorrect in one study [38]. The allocation concealment was not described in 3 studies [33, 36, 38]. In 5 studies [31, 32, 36–38], blinding of participants and personnel was not performed. Blinding of outcome assessment was not mentioned in 3 studies [34, 36, 38].

Four studies were at high risk of small sample bias because the sample size was less than 50 [33, 34, 37, 38]. Two studies had a sample size of between 50 and 200 participants were at moderate risk of small sample bias [35, 36]. Two studies were classified as having a low chance of small study bias because the sample size was more than 200 [31, 32]. A funnel plot was not performed considering less than 10 studies in each comparison.

### Effect of intervention

#### CSI versus wait and see, usual care, and sham intervention

**Fig. 3:**
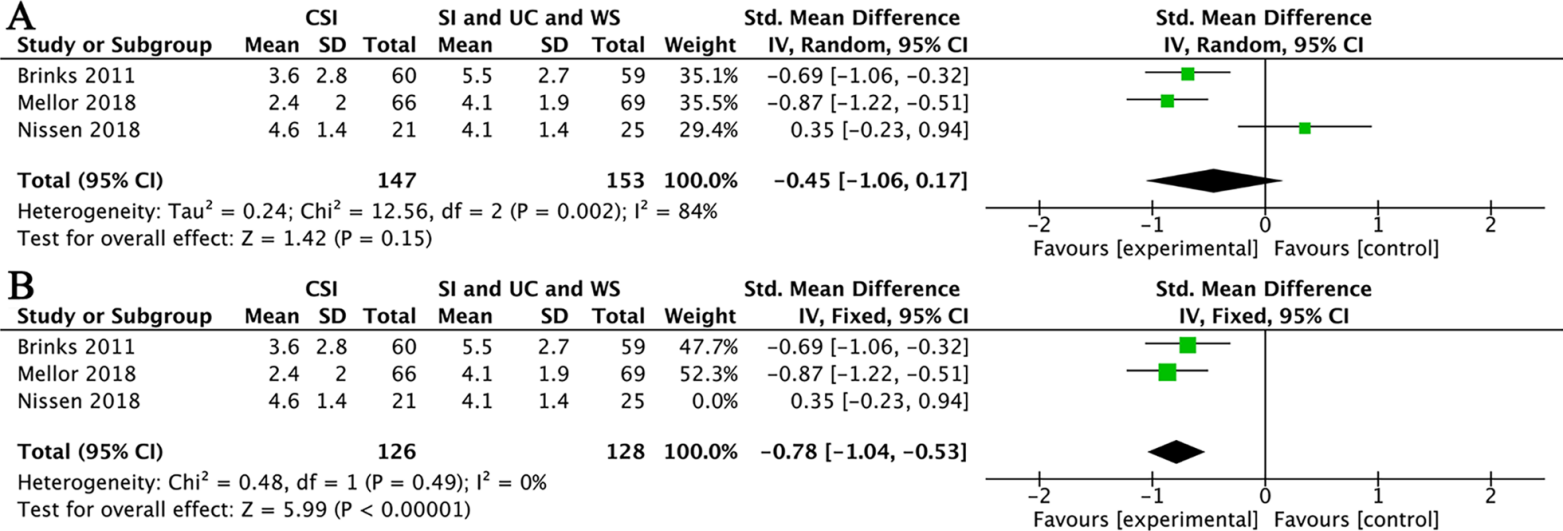
(**A**) CSI versus wait and see, usual care and sham intervention in the short-term pain relief. (**B**) CSI versus wait and see, usual care and sham intervention in the short-term pain relief after removal of one study for sensitivity analysis. Abbreviations: SI, sham intervention; UC, usual care; WS, wait and see

In terms of short-term pain relief, three studies compared CSI and usual care [36], CSI and ‘wait and see’ [31], CSI and sham intervention [34] to observe the effect of CSI in the treatment of GTPS were pooled for meta-analysis. There was no significant difference in favor of CSI (SMD = − 0.45, 95% CI (− 1.06, 0.17); *I*^2^ = 84%; *P* = 0.15) (Fig. 3A). Considering the high risk of small sample bias in one study [34] and the obvious heterogeneity between the included studies (Chi^2^ = 12.56, df = 2 (*P* = 0.002), *I*^2^ = 84%), a sensitivity analysis was performed. There was no significant difference in heterogeneity (Chi^2^ = 0.48, df = 1 (*P* = 0.49), *I*^2^ = 0%) by excluding this study. The results showed that CSI significantly decreased NRS score (SMD = − 0.78, 95% CI (− 1.04, − 0.53); *I*^2^ = 0%; *P* < 0.00001) compared to usual care and ‘wait and see’ in short term (Fig. 3B). There are no data available to analyze short-term function improvement.

**Fig. 4:**
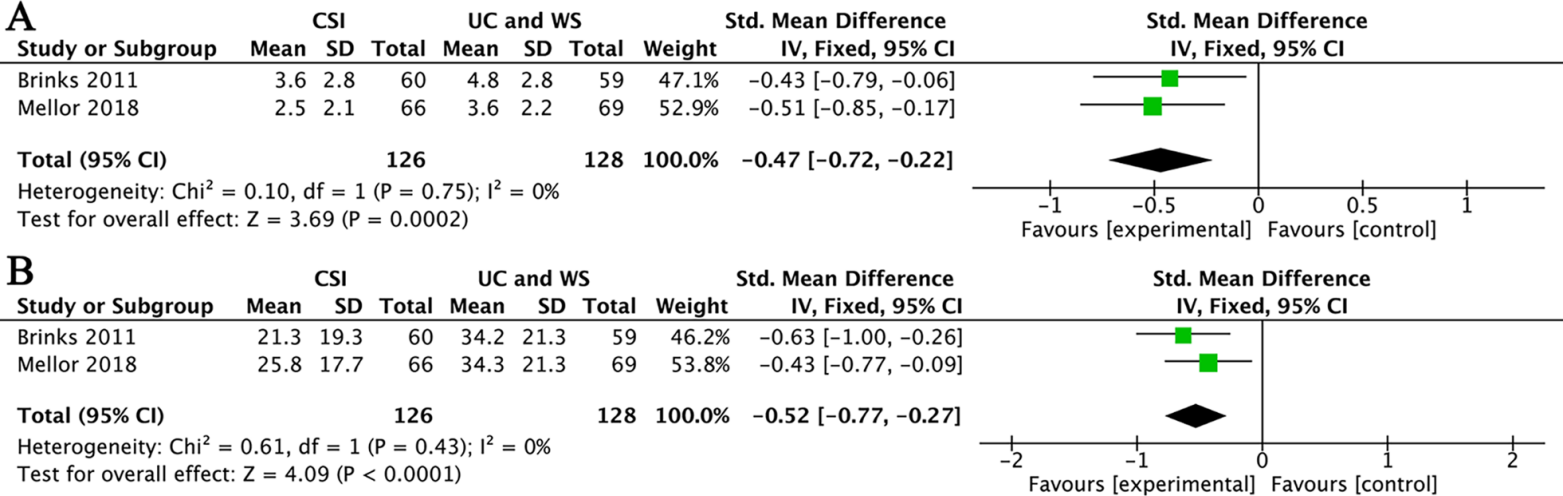
(**A**) CSI versus ‘wait and see’ and usual care in the medium-term pain relief. (**B**) CSI versus ‘wait and see’ and usual care in the medium-term function improvement. Abbreviations: UC, usual care; WS, wait and see

In terms of medium-term pain relief, two studies were pooled for comparison of CSI versus usual care and ‘wait and see’ [31, 36]. Aggregate analysis showed that CSI significantly decreased NRS score (SMD = − 0.47, 95% CI (− 0.72, − 0.22); *I*^2^ = 0%; *P* = 0.0002) in medium term (Fig. 4A). In terms of improving function in the medium term, these two studies were also pooled for analysis. Since these two studies did not use a common scale, one study [36] used the Western Ontario and McMaster University Osteoarthritis Index (WOMAC), so in the other study [31], we chose the lateral hip pain questionnaire (LHPQ) with the same direction (the lower the score, the better the function) to match it for statistical analysis. The data showed that CSI significantly improved the function compared to usual care and ‘wait and see’ in medium term (SMD = − 0.52, 95% CI (− 0.77, − 0.27); *I*^2^ = 0%; *P* < 0.0001) (Fig. 4B).

**Fig. 5:**
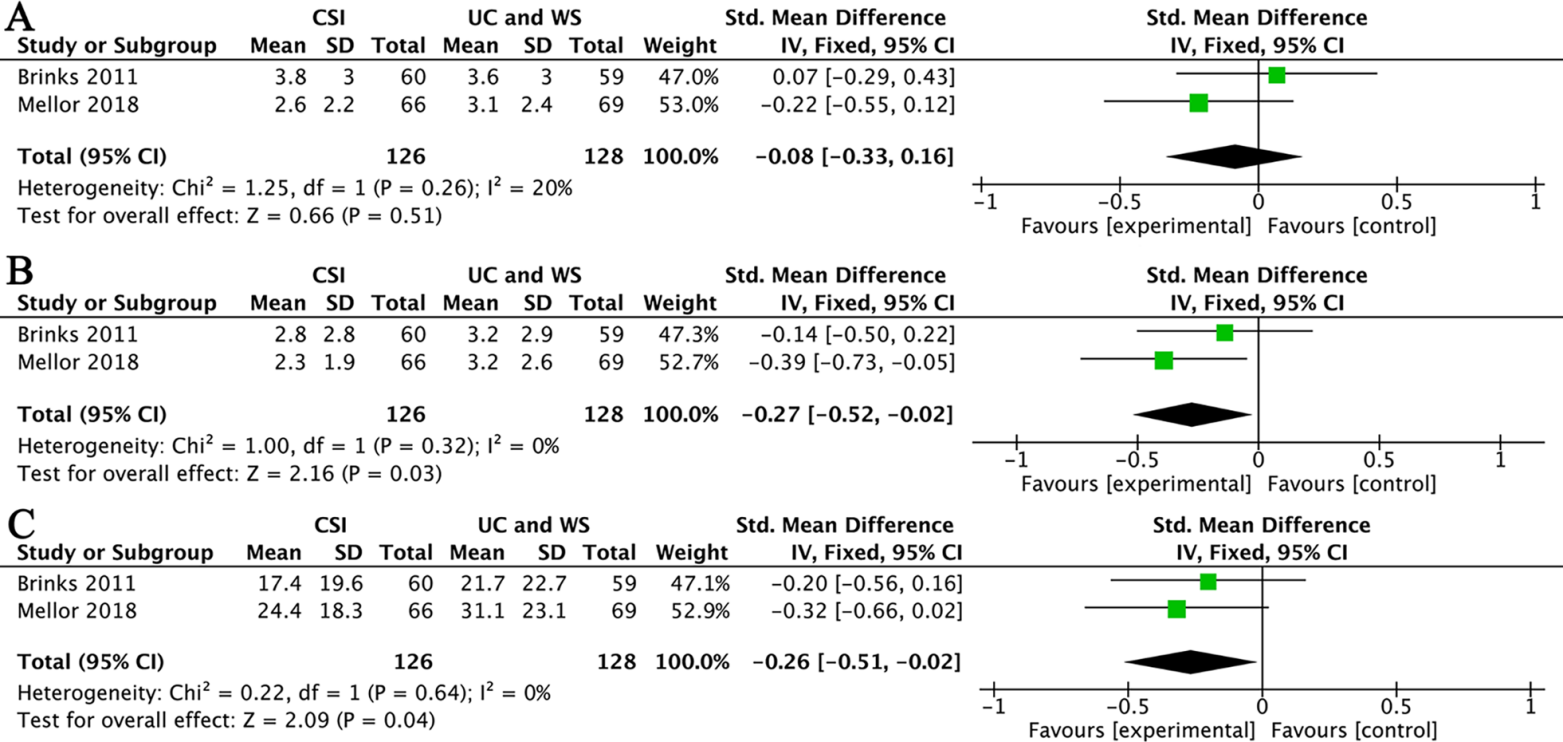
(**A**) CSI versus ‘wait and see’ and usual care in the long-term (6-month) pain relief. (**B**) CSI versus ‘wait and see’ and usual care in the long-term (12-month) pain relief. **(C)** CSI versus ‘wait and see’ and usual care in the long-term (12-month) function improvement. Abbreviations: UC, usual care; WS, wait and see

In terms of long-term pain relief, two studies provided analyzable data for NRS [31, 36]. These two studies have 6-month and 12-month data available. The results showed that CSI did not significantly reduce the pain score compared to usual care and ‘wait and see’ in 6 months (SMD = − 0.08, 95% CI (− 0.33, 0.16); *I*^2^ = 20%; *P* = 0.51) (Fig. 5A), but CSI significantly decreased the pain score in 12 months (SMD = − 0.27, 95% CI (− 0.52, − 0.02); *I*^2^ = 0%; *P* = 0.03) (Fig. 5B). In terms of long-term function improvement, these two studies still have 12-month WOMAC and LHPQ data available for analysis. The data showed that CSI significantly improved the function compared to usual care and ‘wait and see’ in 12 months (SMD = − 0.26, 95% CI (− 0.51, − 0.02); *I*^2^ = 0%; *P* = 0.04) (Fig. 5C).

#### CSI versus exercise

**Fig. 6:**
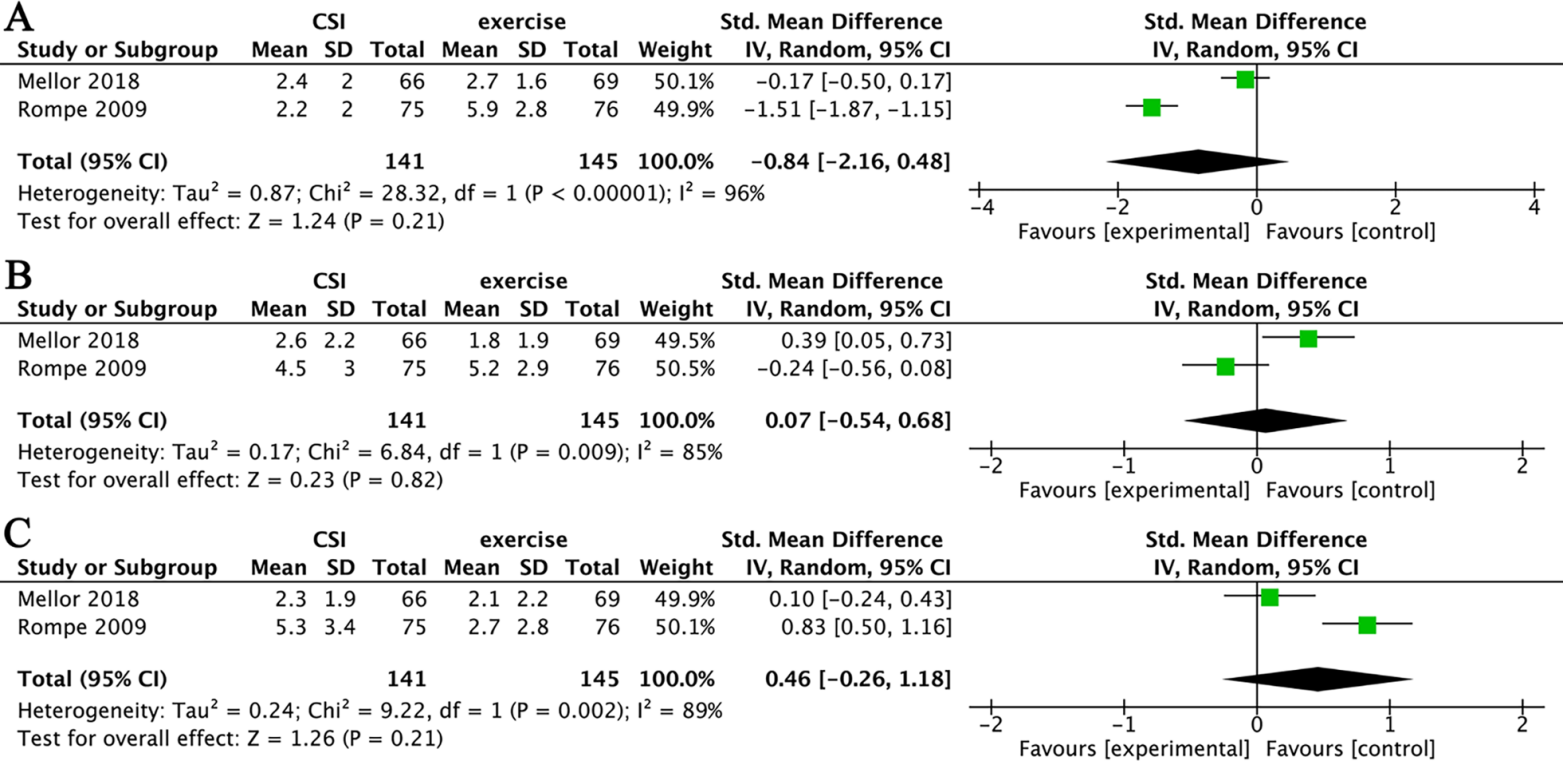
(**A**) CSI versus exercise in the short-term pain relief. (**B)** CSI versus exercise in the long-term (between 3-month and 6-month) pain relief. (**C**) CSI versus exercise in the long-term (more than 12-month) pain relief

In terms of short-term pain relief, two studies compared CSI and exercise were pooled for analysis [31, 32]. Data showed that, compared with exercise, CSI did not significantly decrease NRS score (SMD = − 0.84, 95% CI (− 2.16, 0.48); *I*^2^ = 96%; *P* = 0.21) (Fig. 6A). There are no data available to analyze short-term function improvement.

In terms of medium-term pain and function improvements, there are no data available for analysis.

In terms of long-term pain relief, these two studies have between 3-month and 6-month, and more than 12-month data available for analysis. The results showed that, compared with exercise, CSI did not significantly decrease the NRS score, whether it is between 3 months and 6 months (SMD = 0.07, 95% CI (− 0.54, 0.68); *I*^2^ = 85%; *P* = 0.82) (Fig. 6B) or more than 12 months (SMD = 0.46, 95% CI (− 2.26, 1.18); *I*^2^ = 89%; *P* = 0.21) (Fig. 6C). There are no data available to analyze long-term function improvement.

#### CSI versus PRP

**Fig. 7:**
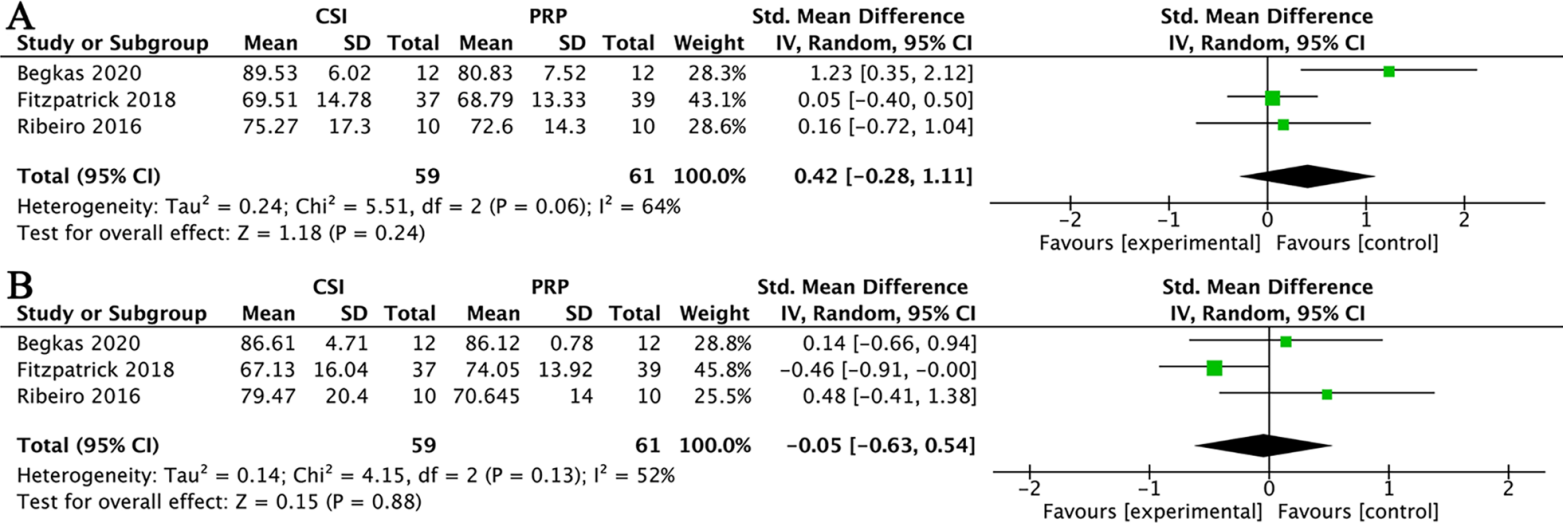
(**A**) CSI versus PRP in short-term function improvement. (**B**) CSI versus PRP in medium-term function improvement

In terms of short-term function improvement, three studies were pooled for comparison of CSI versus PRP [33, 35, 38]. Since these three studies did not use a common scale, so we chose the Harris Hip Score (HHS) in two studies [33, 38], and the modified Harris Hip Score (mHHS) in the other [35]. Data showed that CSI did not significantly improve the function compared with PRP (SMD = 0.42, 95% CI (− 0.28, 1.11); *I*^2^ = 64%; *P* = 0.24) (Fig. 7A). There are no data available to analyze short-term pain relief.

In terms of medium-term function improvement, these three studies were still included in the analysis [33, 35, 38]. The data of HHS and mHHS were analyzed, and the results showed that there was no significant difference in the functional improvement between CSI and PRP (SMD = − 0.05, 95% CI (− 0.63, 0.54); *I*^2^ = 52%; *P* = 0.88) (Fig. 7B). There are no data available to analyze medium-term pain relief.

In terms of long-term function improvement, one studies [38] compared CSI versus PRP with a statistically significant (MD = − 38.25, 95% CI (− 44.56, − 31.94)) in favor of PRP. There are no data available to analyze long-term pain relief.

Significant heterogeneities were observed in these studies, and sensitivity analysis also showed no significant difference in short-term and medium-term function improvements.

#### CSI versus dry needling

In terms of short-term pain and function improvements, one study [37] compared CSI and dry needling, and there was no significant difference in reducing the NRS score (MD = 1.1, 95% CI (− 0.63, 2.83)) and improving the patient-specific functional scale (PSFS) (MD = − 1.2, 95% CI (− 2.68, 0.28)).

There are no data available to analyze the medium-term and long-term pain and function improvements.

#### CSI versus ESWT

In terms of short-term pain relief, one study [32] compared CSI versus ESWT with a statistically significant (MD = − 3.4, 95% CI (− 4.34, − 2.46)) in favor of CSI. There are no data available to analyze the short-term function improvement.

There are no data available to analyze the pain and function improvements in medium term.

In terms of long-term pain relief, one study [32] compared CSI with ESWT, and data at two time points in 4 months and 15 months are available. The results showed that, compared with CSI, ESWT significantly decreased NRS score in 4 months (MD = 1.3, 95% CI (0.44, 2.16)) and 15 months (MD = 2.9, 95% CI (1.87, 3.93)). There are rno data available to analyze function improvement in long term.

## Discussion

In the management of GTPS, CSI is a commonly used treatment option [6]. However, the role of CSI in GTPS is still controversial, CSI was recommended up to 2–3 times per patient per year by 40% of physiotherapists, while CSI was rare or not recommended by 60% of physiotherapists [39]. Some RCT and observational studies suggested that CSI was effective in the short term [31, 36, 40], while another RCT study showed that CSI was not superior to placebo groups in short term [34]. The efficacy of CSI is also inconsistent in the long-term follow-up [31, 36]. At the same time, CSI has been compared with PRP, ESWT, dry needling, and exercise in many studies [31–33, 37]. So, we performed this review to comprehensively evaluate the therapeutic effect of CSI on GTPS.

Previous systematic reviews of CSI in GTPS have stated that CSI has a short-term benefit, but not in a long term [10, 16]. In this review, due to the limited number of selected articles, the three studies compared CSI with sham intervention, usual care, and ‘wait and see’ were pooled for meta-analysis to evaluate the therapeutic effect of CSI on GTPS [31, 34, 36]. The pooled standardized mean difference suggested that CSI had no significant effect on relieving pain in short term, but after excluding the small sample size article [34] comparing CSI and sham intervention through sensitivity analysis, the results showed that CSI had a significant effect in short-term pain relief. Two studies have compared CSI to usual care and ‘wait and see’ in medium term and long term [31, 36], and the pooled data suggested that CSI had a significant effect in medium-term pain and function improvements, and the effects diminished at 6 months, but it still had a significant effect compared to the control group at 12 months. So, in terms of short term and medium term (within 3 months) efficacy, we are consistent with the results of previous systematic reviews, but in terms of long-term efficacy at 12 months, our meta-analysis suggests that CSI is still effective, which is different from previous cognition. Therefore, the therapeutic effect of CSI on GTPS is still controversial, and placebo-controlled studies with larger sample sizes are needed in the future to prove its effect.

Two studies have compared CSI to exercise [31, 32], and the pooled data suggest that there is no significant difference in short-term and long-term pain relief. These results suggest that CSI is not superior to exercise, and there has been no meta-analysis to evaluate them in the past. Due to significant heterogeneity, no definitive conclusion can be drawn.

Three studies have compared CSI to PRP [33, 35, 38], and the pooled data suggest that there is no significant difference in short-term and medium-term function improvements. In long-term function improvement, only one study has compared CSI to PRP [38], and this single study suggests that CSI was inferior to PRP at 6 months. The existence of significant heterogeneity makes the results less credible. This heterogeneity might mainly come from the small sample size, different scales, different corticosteroid drugs, and different literature quality.

One study has compared CSI to dry needling [37], and this single study suggests that CSI was not superior to dry needling in terms of short-term effects. Only one study suggests this result, and at the same time it is a small sample size study, so the effect of CSI over dry needling cannot be clearly stated.

CSI has been compared with ESWT in a single study [32], and this study suggests CSI was superior to ESWT in alleviating short-term pain, but inferior to ESWT in long-term pain relief. Considering the limited included articles and possible performance bias, the effect of CSI over ESWT cannot be definitively stated.

Admittedly, some obvious limitations presented in this review. First, only English language literature were included, which may introduce potential publication bias. Second, only 8 studies were included in our review, only 1–3 studies were classified into each comparison group, and 4 of 8 studies were small sample size studies, so publication bias might exist. But publication bias was not evaluated because there were less than 10 studies in each comparison group. Third, different outcome measures were used in the included studies, challenging our review, even if we used SWD. Standardization of validated and reliable outcome measures will improve the quality of systematic reviews [41]. Recently, VISA-G has been proven to be an effective and reliable scale in assessing GTPS-related disability [42]. However, in this review, only one study used this scale. Fourth, different control interventions were classified into one category to analyze the therapeutic effect of CSI on GTPS. Control interventions included usual care, ‘wait and see,’ and sham intervention. Only one study compared CSI to sham intervention, but the result was negative, which is different from the results of other studies.

## Conclusions

This is the first meta-analysis to evaluate the efficacy of CSI relative to other methods for the treatment of GTPS. This systematic review shows that there is a lack of high-quality, large-sample RCTs on the CSI treatment GTPS. Based on limited evidence, this review found: First, CSI may be superior to usual care and ‘wait and see’ in short-term and medium-term pain relief and medium-term function improvements, and this effect may last for more than 1 year; second, CSI may be not superior to exercise, whether it is short-term or long-term pain relief; third, CSI may be not superior to PRP in short-term and medium-term function improvements and may even be inferior to PRP in long-term function improvement; fourth, CSI may be not superior to dry needling in short-term pain and function improvements; fifth, CSI may be superior to ESWT in short-term pain relief, but inferior to ESWT in long-term pain relief. Findings indicate that high-quality, large-sample studies are needed in the future to clarify the role of CSI in GTPS treatment, especially compared with placebo.

## Supplementary Information

**Additional file 1**. PRISMA checklist

## Data Availability

All data generated or analyzed during this study are included in this published article and its Additional files.

## Abbreviations

CI: Confidence intervals
CSI: Corticosteroid injection
d: Day
ESWT: Extracorporeal shockwave therapy
EQ-5D: European quality of life-5D questionnaire
F: Female
FEPS: Facial expressions pain scale
GTPS: Greater trochanteric pain syndrome
HHS: The Harris Hip Score
LHPQ: Lateral hip pain questionnaire
M: Male
m: Month
MD: Mean difference
mHHS: The modified Harris Hip Score
NRS: Numerical rating scale
PSFS: Patient-specific functional scale
VAS: Visual analogue scale
PASS: Patient acceptable symptom state
PSEQ: Pain self-efficacy questionnaire
PCS: Pain catastrophising scale
PHQ-9: Patient health questionnaire 9
RCT: Randomized controlled trail
SD: Standard deviation
SMD: Standardized mean difference
VISA-G: Victorian Institute of Sport Assessment—gluteal tendinopathy
w: Week
WOMAC: The Western Ontario and McMaster University Osteoarthritis Index
